# Low expression of IL-18 and IL-18 receptor in human skeletal muscle is associated with systemic and intramuscular lipid metabolism—Role of HIV lipodystrophy

**DOI:** 10.1371/journal.pone.0186755

**Published:** 2018-01-17

**Authors:** Birgitte Lindegaard, Thine Hvid, Helene Wolsk Mygind, Ole Hartvig-Mortensen, Thomas Grøndal, Julie Abildgaard, Jan Gerstoft, Bente Klarlund Pedersen, Marcin Baranowski

**Affiliations:** 1 The Centre of Inflammation and Metabolism and The Centre of Physical Activity Research, Rigshospital, Copenhagen, Denmark; 2 The Department of Infectious Diseases, Rigshospitalet, Copenhagen, Denmark; 3 The Department of Lung- and Infectious Diseases, Nordsjællands Hospital, Hillerød, Denmark; 4 Department of Biomedical Science, University of Copenhagen, Denmark; 5 Department of Physiology, Medical University of Bialystok, Bialystok, Poland; Wake Forest University School of Medicine, UNITED STATES

## Abstract

**Introduction:**

Interleukin (IL)-18 is involved in regulation of lipid and glucose metabolism. Mice lacking whole-body IL-18 signalling are prone to develop weight gain and insulin resistance, a phenotype which is associated with impaired fat oxidation and ectopic skeletal muscle lipid deposition. IL-18 mRNA is expressed in human skeletal muscle but a role for IL-18 in muscle has not been identified. Patients with HIV-infection and lipodystrophy (LD) are characterized by lipid and glucose disturbances and increased levels of circulating IL-18. We hypothesized that skeletal muscle IL-18 and IL-18 receptor (R) expression would be altered in patients with HIV-lipodystrophy.

**Design and methods:**

Twenty-three HIV-infected patients with LD and 15 age-matched healthy controls were included in a cross-sectional study. Biopsies from the vastus lateralis muscle were obtained and IL-18 and IL-18R mRNA expression were measured by real-time PCR and sphingolipids (ceramides, sphingosine, sphingosine-1-Phosphate, sphinganine) were measured by HPLC. Insulin resistance was assessed by HOMA and the insulin response during an OGTT.

**Results:**

Patients with HIV-LD had a 60% and 54% lower level of muscular IL-18 and IL-18R mRNA expression, respectively, compared to age-matched healthy controls. Patients with HIV-LD had a trend towards increased levels of ceramide (18.3±4.7 versus 14.8±3.0,p = 0.06) and sphingosine (0.41±0.13 versus 0.32±0.07, and lower level of sphinganine (p = 0.06). Low levels of muscle IL-18 mRNA correlated to high levels of ceramides (r = -0.31, p = 0.038) and sphingosine-1P (r = -0.29, p = 0.046) in skeletal muscle, whereas such a correlation was not found in healthy controls. Low expression of IL-18 mRNA in skeletal muscle correlated to elevated concentration of circulating triglycerides (R_p_ = -0.73, p<0.0001). Neither muscle expression of IL-18 mRNA or ceramide correlated to parameters of insulin resistance.

**Conclusion:**

IL-18 (mRNA) in skeletal muscle appears to be involved in the regulation of intramuscular lipid metabolism and hypertriglyceridemia.

## Introduction

The cytokine interleukin (IL)-18 is a member of the IL-1 family and has been identified as a cofactor that, together with IL-12, stimulates production of interferon gamma [1]. IL-18 is widely expressed in many mammalian cells/tissues including liver, adipose tissue and skeletal muscle [2–4] [5]. IL-18 is best known for its role in inflammation, whereby pro-inflammatory stimuli such as lipopolysaccharide, and tumor necrosis factor (TNF)-α leads to caspase-1 mediated cleavage of pro-IL-18 into mature IL-18. IL-18 is synthesized as an inactive precursor molecule (Pro-IL-18) that lacks a signal peptide and requires cleavage into a mature, active cytokine IL-18 that is then secreted from the cell [6].

IL-18 can then signal via a heterodimer of the transmembrane IL-18 receptors (α and β), and via a toll like receptor signaling cascade ultimately leading to the activation of nuclear factor κB (NFκB) and subsequent regulation of gene transcription [7]. Additional signaling pathways of IL-18 also exist, including activation of phosphatidylinositol-3 kinase (PI3K)/Akt [8,9], signal transducer and activator of transcription 3 (STAT 3) [10], mitogen-activated protein kinases (MAPK) [8,10], and c-Jun NH_2_-terminal kinase (JNK) [9,11] which are all implicated in energy metabolism.

As IL-18 plays a role in inflammation it is not surprisingly that circulating IL-18 levels are elevated in human obesity [12] and in patients with type 2 diabetes [13,14]. IL-18 is expressed in adipose tissue [15,16] especially in visceral adipose tissue [17,18].

Paradoxically, IL-18 has also been found to be directly involved in the regulation of lipid and glucose metabolism as mice lacking whole-body IL-18 signalling become obese and insulin resistant, [19,20]. In a previous study, we demonstrated that IL-18 receptor deficient mice display obesity, insulin resistance, impaired fat oxidation and ectopic lipid deposition in liver and skeletal muscle [21]. Moreover, administration of IL-18 to whole muscle strips ex vivo increased AMPK signaling and increases fat oxidation, and *in vivo* electroporation of IL-18 into skeletal muscle results in increased AMPK signaling and expression of mitochondrial genes in skeletal muscle and concomitantly inhibits high fat diet-induced-weight gain, suggesting that IL-18 is increasing skeletal muscle fat oxidation via AMPK and oppose ectopic lipid accumulation [21]. Taken together, in the progression toward obesity, there is continual production of IL-18 to oppose ectopic lipid accumulation [22]. However, the cellular origin of IL-18 remains enigmatic, although it has been shown that IL-18 released from adipose tissue is not produced by the adipocytes themselves [16,23]. Given that IL-18 is expressed in human skeletal muscle and exerts its effect on lipid metabolism in skeletal muscle *ex vivo* and *in vivo* in rodents, it suggest that IL-18 is regulated in skeletal muscle and associated to lipid metabolism. However, the role of IL-18 in skeletal muscle in humans have never been investigated.

A syndrome of lipodystrophy, characterised by subcutaneous fat loss, and a relative increase in central fat accumulation, was previously seen in patients with HIV, when treated with a combination of antiretroviral therapy, including thymidine-nucleoside analogues [24–26]. Lipodystrophy is mostly caused by antiretroviral induced adipose tissue dysfunction [27,28] and is associated with impaired fat oxidation [29,30], ectopic lipid deposition in muscle and liver [24,30–32], and mitochondrial dysfunction in skeletal muscle [33] leading to dyslipidemia and insulin resistance [24,25]. Elevated circulating levels of IL-18 are observed in HIV-infected patients and especially those with lipodystrophy [34,35]. The increase in circulating IL-18 is associated to the fat redistribution and in part derived from subcutaneous adipose tissue [36,37].

The ectopic accumulation of lipids in the skeletal muscle is closely linked with insulin resistance [38]. In the recent years it is found that it is not the total amount of lipid but the lipid intermediates causing insulin resistance. The lipid intermediates account for sphingolipids, e.g. ceramides, sphingosine, sphingoanine, sphingosine-1-P, and diacylglycerol (DAG) [39]. Especially, ceramides [40] and DAG have been supposed to induce insulin resistance [38]. Besides antagonize insulin signalling recent data also shows that ceramides impair mitochondrial functions [41].

In this study, we included material from a cohort of HIV patients with lipodystrophy [42] in order to obtain more information about the metabolic role of IL-18 in humans. Patients with HIV-lipodystrophy share some of the same metabolic disturbances as mice lacking IL-18 signalling but paradoxically the systemic levels of IL-18 are increased in those patients. We therefore aimed at determining whether muscle IL-18 mRNA and IL-18 receptor mRNA are altered, either increased or decreased, in these patients. Given the strong link between ceramides and insulin resistance [38] and mitochondrial function [41], we further studied the possible association of muscle IL-18 mRNA with ceramides and other sphingolipids.

## Patients and methods

### Patients and controls

A group of 23 HIV-infected men were recruited from the outpatient clinic of the Department of Infectious Disease, Rigshospitalet in Copenhagen. These subjects have been included in a former study and the inclusions of the subjects are described therein [42]. In short, LD was defined clinically by physical examination of peripheral lipoatrophy (defined by the presence of peripheral lipoatrophy with at least one moderate sign of fat loss in face, arms, buttocks, or legs based on a physical examination by a single investigator (BL) using a validated questionnaire developed by Carr et al [43]). All patients were on a stable and effective nucleoside analogue based antiretroviral therapy with no changes during the preceding 8 weeks.

Two groups of healthy controls were included: Fifteen age-matched HIV-negative healthy men served as controls for RT-PCR data (Group 1). These subjects have also been included in the before mentioned study [42]. But as muscle tissue for measurement of sphingolipids from healthy controls in group 1 were available for only two healthy subjects, 17 new healthy age-matched control subjects were included (Group 2). Demographic data were collected for each patient: age, duration of HIV infection, duration and types of all antiretroviral therapy, weight, height, CD4 count, HIV-RNA copies. Inclusion criteria: no signs of ongoing infections; fasting glucose < 7 mmol/L and 120 min glucose after an OGTT < 11.1 mmol/L, no dyslipidemia (triglycerides >1.7 mmol/L and/or HDL-cholesterol <0.9 mmol/L); suppressed viral load (<20 copies/mL). Exclusion criteria: Severe cardiovascular diseases; arthritis; severe neuropathy; hepatitis C; opportunistic infections that required hospitalisation within the last 6 weeks; diabetes (fasting glucose ≥7 mmol/L or 2-hrs. glucose >11 mmol/L after an OGTT); concurrent therapy with antidiabetic agents, anticoagulant or any hormones.

Written informed consent was obtained from all subjects according to the requirements from the local ethical committee and the Helsinki Declaration II, and the approval from the local ethical committee (KF 01269485): The Ethics Committee of Copenhagen and Frederiksberg) was obtained.

### Biochemical measurements

Peripheral blood samples were obtained at 8 AM after an overnight fasting. Measurements of total cholesterol (mmol/L), HDL-cholesterol (mmol/L), LDL-cholesterol (mmol/L), triglycerides (mmol/L), plasma glucose (mmol/L) and insulin (pmol/L), were determined immediately using routine methods.

CD4 cell counts were calculated by flowcytometry and HIV-RNA copies were measured by the Amplicor HIV Monitor (Roche Molecular Systems, Branchburg, NJ) (lower limit of dectection: 20 copies/ml).

### Body composition analysis

Fat and fat-free tissue masses for whole body, trunk and extremities were measured using dual-energy X-ray absorptiometry (DXA) scanner (Lunar Prodigy, GE Medical Systems Wisconsin, USA, version 8.8) [43]. Whole-body and regional fat measurements (trunk and extremities) were determined as previously described [44].

## Maximal oxygen consumption (VO_2max_)

An incremental exercise to volitional fatigue was performed between 0800 h and 1000 h on a cycle ergometer (Monark 839E, Monark Ltd, Varberg, Sweden). Maximal oxygen consumption (VO_2max_) was measured with an indirect calorimetric system (Moxus modular VO2 system, AEI Technologies, Pittsburgh, PA) using a 2-way non-rebreathing valve (Hans Rudolph, Inc. Kansas City, Missouri) which recorded data every 15 seconds. Based on the pre-VO_2max_ test a protocol was designed in order to reach VO_2max_ within 8–12 min of exercise start [45]. Exhaustion was defined by two of the following: respiratory exchange ratios >1.10, VO_2_ reached a plateau and/or rpm <60 in more than 10 sec.

### Insulin sensitivity

Insulin resistance was assessed from several measurements: fasting plasma insulin, homeostasis model (HOMA-IR) [46] and area under the curve (AUC) for the insulin concentration during a 75-g oral glucose tolerance test (OGTT)

### Muscle tissue biopsies

Muscle tissue biopsies were obtained after an overnight fast by use of the percutaneous biopsy technique with suction from the quadriceps muscle under local anaesthesia with 2% lidocaine. Muscle tissue was immediately frozen in liquid nitrogen and stored at –80°C until analysed.

#### RNA extraction

RNA was extracted using Trizol^™^ (Life Technologies) according to manufacturer’s protocol. In short, 1 ml of Trizol^™^ was added to 20–30 mg of muscle tissue and homogenized using a Polytron (PT-MR2100, Kinematica) on setting 25–30 for 20–30 s and placed on ice. All samples were added 100 μl of chloroform, shaken vigorously and incubated for 5 min on ice. Samples were spun at 12000 g for 15 min at 4°C, and the upper aqueous phase was placed in a fresh eppendorph tube. The same volume of isopropanol was added and samples were placed at –20°C for 1 hour followed by centrifugation at 12000 g for 15 min at 4°C. The resulting RNA pellet was washed with 75% ethanol in DEPC-treated water and spun at 6000 g for 10 min at 4°C. The pellets were dissolved in DEPC-treated water.

#### Reverse transcription

One μg of total RNA was reverse transcribed in a 50-μl reaction according to manufacturer's protocol (Applied Biosystems, Taqman^™^ reverse transcription reagents) with the use of random hexamer primers. The reactions were run in a Perkin Elmer GeneAmp PCR system 9700 with conditions at 25°C for 10 min, 48°C for 30 min, and 95°C for 5 min.

#### Analysis of gene expression levels in muscle tissue

Samples were analyzed for IL-18 and IL-18 receptor mRNA levels by real-time PCR using an ABI PRISM 7900 sequence detector (PE Biosystems). The gene expression levels were normalized to the housekeeping gene GAPDH (obtained from Applied Biosystems). Human IL-18 (Hs00155517_m1) and human IL-18 receptor (Hs00187256_m1) primers and Taqman^®^ probe were obtained from Applied Biosystems. All reactions were run in triplicates.

Data were quantitated and normalized using the standard curve method.

#### Sphingolipid analysis

The content of S1P, SA1P, sphingosine, sphinganine, and ceramide was determined as described previously in detail (Knapp et al. 2013). Briefly, lipids were extracted from samples in the presence of internal standards (10 pmol of C_17_-sphingosine and 30 pmol of C_17_-S1P, Avanti Polar Lipids, Alabaster, AL). An aliquot of the lipid extract was transferred to a fresh tube with pre-added 40 pmol of N-palmitoyl-D-erythro-sphingosine (C17 base) (a kind gift of Dr Z. Szulc, Medical University of South Carolina) as an internal standard, and then subjected to alkaline hydrolysis to deacylate ceramide to sphingosine. The amount of S1P and SA1P was determined indirectly after dephosphorylation to sphingosine and sphinganine, respectively, with the use of alkaline phosphatase (bovine intestinal mucosa, Sigma). Free sphingosine and sphinganine, dephosphorylated sphingoid bases, and sphingosine released from ceramide were then converted to their o-phthalaldehyde derivatives and analyzed using a HPLC system (ProStar, Varian Inc., Palo Alto, CA) equipped with a fluorescence detector and C18 reversed-phase column (Varian Inc. OmniSpher 5, 4.6×150mm). The isocratic eluent composition of acetonitrile (Merck, Darmstadt, Germany): water (9:1, v/v) and a flow rate of 1 ml/min were used. Column temperature was maintained at 30°C [47].

### Statistical analysis

Statistical calculations were performed using SAS 9.1 (USA). Data are presented as means +/- SD. *P* < 0.05 was considered significant in all analyses. Parameters between patients with HIV-lipodystrophy and healthy controls were compared with a Mann-Whitney test. Pearson's correlations were used to examine the relationship between mRNA expression in muscle, sphingolipid in muscle and markers of insulin sensitivity, as well as with anthropometric parameters.

## Results

### Baseline characteristics

Demographic data, blood biochemistry and body composition appear in Table 1.

**Table 1:** Baseline characteristics of patients and healthy controls.

	Group 1		Group 2	
Variab	Healthy controls (n = 15)	Patients with HIV-LD (n = 23)	Healthy controls (n = 17)	Patients with HIV-LD (n = 14)§
Age (years)	47.5 (6.1)	47.9 (9.5)	46.5 (6.0)	48.3 (9.7)
Duration of HIV infection (years)		15.6 (9.6)		13.9 (7.0)
Duration of antiretroviral therapy (years)		10.3 (4.3)		8.9 (3.8)
CD4+ cell (cells/μl)		558 (208)		550 (250)
LogHIV-RNA (copies/ml)		1.33 (0.12)		1.32 (0.11)
**Antiretroviral use**				
Current Tymidine-NRTI use, No. (%)		11 (47.8)		7 (50.0)
Current PI use, No. (%)		13 (56.7)		8 (57.1)
Current NNRTI use, No. (%)		11 (47.8)		7 (50.0)
Physical activity parameters				
VO_2max_ (LO_2_/min)	2.5 (0.6)	2.3 (0.5)	3.4 (0.8)	2.4 (0.5)***
Body composition				
Body-mass index (kg/m^2^)	23.7 (1.9)	23.7 (2.9)	23.3 (2.1)	24.1 (3.0)
Weight (kg)	76.9 (7.4)	73.6 (11.2)	79.4 (9.0)	75.8 (11.1)
Waist (cm)	90 (5.7)	93.6 (6.4)	91.1 (7.9)	94.2 (7.2)
Waist-to.hip ratio	0.94 (0.03)	1.01 (0.04)	0.91 (0.05)	1.02 (0.04)
Fat mass (kg)	15.7 (4.4)	13.8 (5.3)	16.2 (6.4)	15.0 (5.3)
Trunk fat mass (kg)	8.9 (3.0)	9.8 (3.9)	9.1 (4.0)	10.9 (4.0)
Trunk fat percentage (%)	56.1(5.2)	71.2 (6.2) ****	55.3 (5.1)	72.5 (5.8)****
Limb fat mass (kg)	6.2 (1.5)	3.5 (1.6)****	6.6 (2.5)	3.6 (1.5)***
Limb fat percentage (%)	40.2 (4.9)	25.1 (6.1) ****	41.5 (4.2)	24.0 (5.7)****
Trunk-to-limb fat ratio	1.4 (0.29)	3.09 (1.17)*	1.4 (0.25)	3.3 (1.3)***
Lean mass (kg)	58.2 (5.2)	57.0 (6.8)	61.0 (5.4)	57.9 (6.2)
**Metabolic parameters**				
Total-cholesterol (mmol/L)	4.63 (0.64)	5.5 (0.9)**	4.81 (0.65)	5.8 (0.7)***
HDL-C (mmol/L)	1.51 (0.32)	1.23 (0.52)*	1.41 (0.32)	1.21 (0.36)
LDL-C (mmol/L)	3.3 (0.6)	3.7 (0.9)	3.14 (0.74)	3.94 (0.81)**
Triglycerides (mmol/L)	0.76 (0.24)	2.55(1.43)****	0.96 (0.26)	2.88 (1..34)****
Glucose (mmol/L)	5.2 (0.3)	5.4 (0.6)	5.0 (0.1)	5.4 (0.7)
Insulin (pmol/L)	25 (8.9)	52 (25)****	28.4 (9.9)	56.5 (28.2)***
HOMA-IR	0.99 (0.37)	2.2 (1.4)****	1.3 (0.14)	2.5 (1.5)
Glucose area under the curve (mmol/Lmin)	670 (126)	826 (200)*	654 (153)	779 (176)
Insulin area under the curve (pmol/Lmin)	23505 (10598)	52360 (31017)**	18436 (10510)	45388 (37026)**

Two groups of healthy men were included due to lack of muscle tissue. Group 1 served as controls for the RT-PCR data. Group 2 served as controls for the sphingolipid analysis.

^§^ Fourteen patients with HIV-LD are a part of the 23 patients with HIV-LD in group 1.

Data are presented as mean (SD). PI, protease inhibitor; NRTI, nucleoside reverse transcriptase inhibitor; NNRTI, non- nucleoside reverse transcriptase inhibitor. HOMA-IR, homeostatic model assessment for insulin resistance.

*P < 0.05;

** *P* < 0.01;

***P < 0.001,

****P < 0.0001 by *t*-test comparing patients with HIV-LD and healthy controls within each cohort.

The healthy men and the patients with HIV-lipodysstrophy were age-matched. Two groups of healthy men were included due to lack of muscle tissue. Group 1 served as controls for the RT-PCR data. Group 2 served as controls for sphingolipid analysis.

In group 1, patients were matched based on their VO_2_ max. In group 2 we also tried to match HIV patients with their control based on physical activity, but at the end of the inclusion the HIV patients had lower levels of VO_2_ max compared with controls (Table 1).

Patients with HIV-lipodystrophy were characterised by reduced total limb fat mass, increased percentage of trunk fat mass, reduced percentage of limb fat mass and increased trunk-to-limb fat mass, indicating fat redistribution, compared to healthy controls. No differences were found regarding BMI, total fat mass, trunk fat mass or lean body mass.

Fasting triglycerides and total-cholesterol levels were higher in patients with HIV-lipodystrophy and so were fasting insulin, HOMA-IR and the insulin response during an OGTT when compared to control subjects from both group 1 and 2, indicating insulin resistance in patients with HIV-lipodystrophy (Table 1).

As previously demonstrated, plasma IL-18 was increased in patients with HIV-lipodystrophy compared to healthy controls (247 pg/ml (98) vs 199 pg/ml (102), p<0.05).

### IL-18 and IL-18 receptor mRNA expression in skeletal muscle

**Fig 1:**
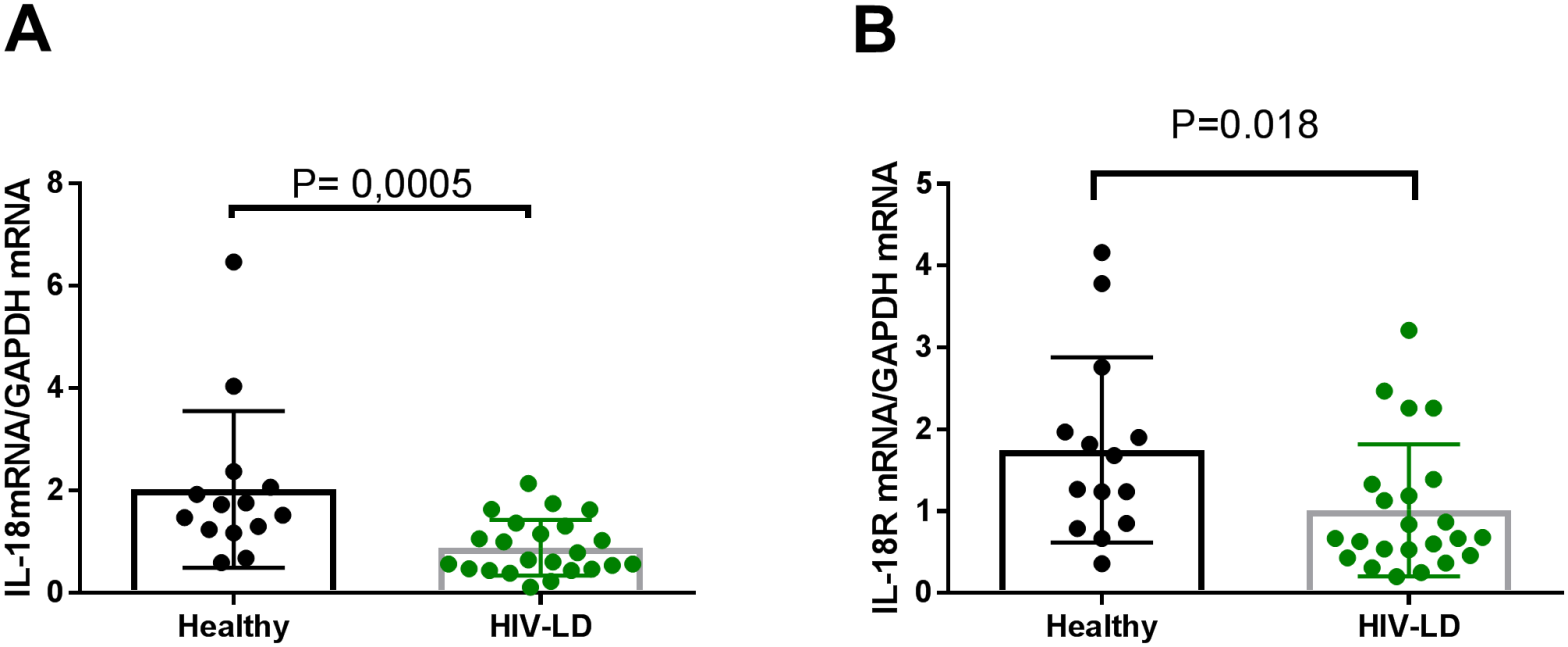
Patients with HIV-lipodystrophy have reduced levels of IL-18 mRNA and IL-18 receptor mRNA in skeletal muscle. (A) mRNA expression of IL-18 in skeletal muscle. In the healthy control group 2 subjects had very high levels of IL-18 mRNA levels. Even if those two subjects were deleted the difference between healthy controls and patients with HIV-Lipodystrophy were still high significant p = 0.003). (B) mRNA expression of IL-18 receptor in skeletal muscle. The levels of IL-18 mRNA and IL-18 receptor mRNA were calculated with GAPDH as a housekeeping gene. In the dot plots data for each subjects are given and the line represent means and SD. * P<0.05 and ***P<0.001 for healthy vs HIV-lipodystrophy patients.

IL-18 mRNA (Fig 1A) and IL-18 receptor mRNA (Fig 1B) expression were reduced by 60% and 54%, respectively, in skeletal muscle in patients with HIV-lipodystrophy compared to healthy controls. (IL-18 mRNA: p = 0.0005 IL-18 receptor mRNA: p = 0.018). In the healthy control group, two subjects had very high levels of IL-18 mRNA. If these two subjects were excluded, IL-18 mRNA expression was still significantly lower in patients with HIV-lipodystrophy compared to healthy controls (p = 0.003) (data not shown).

### Sphingolipid in skeletal muscle

**Fig 2:**
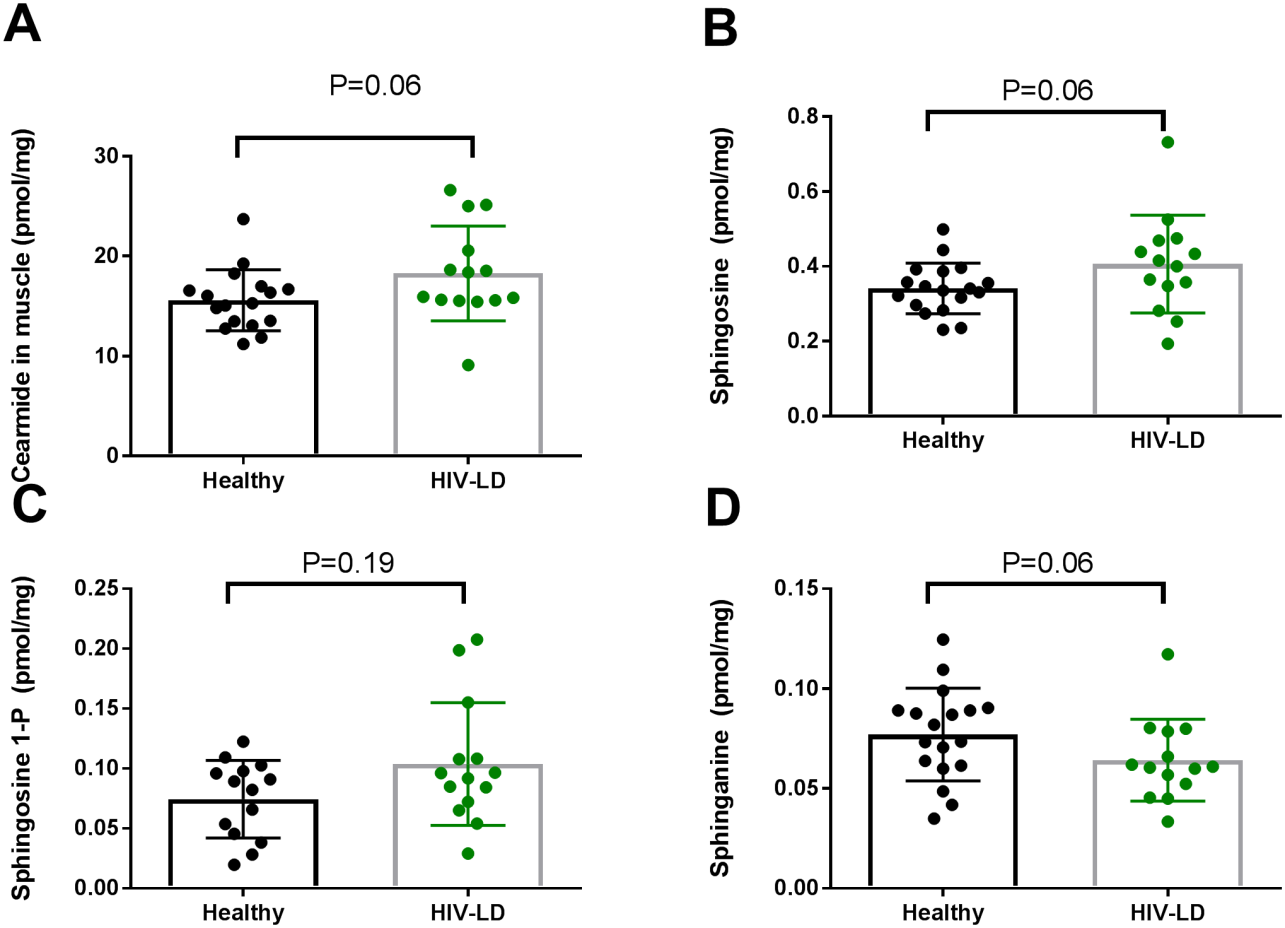
The sphingolipids ceramide and sphingosine tended to be increased in vastus lateralis muscle from patients with HIV-lipodystrophy. In the dot plots data for each subjects are given and the line represent means and SD.

The sphingolipids ceramide (Fig 2A) and sphingosine (Fig 2B) content in muscle tended to be elevated in patients with HIV-lipodystrophy compared to healthy controls (P = 0.06).

The sphinganine content in muscle tended to be lower in patients with HIV-lipodystrophy compared to healthy controls (Fig 2D, p = 0.06)

### Relationship between IL-18 mRNA and sphingolipid in skeletal muscle and circulating lipids

**Fig 3:**
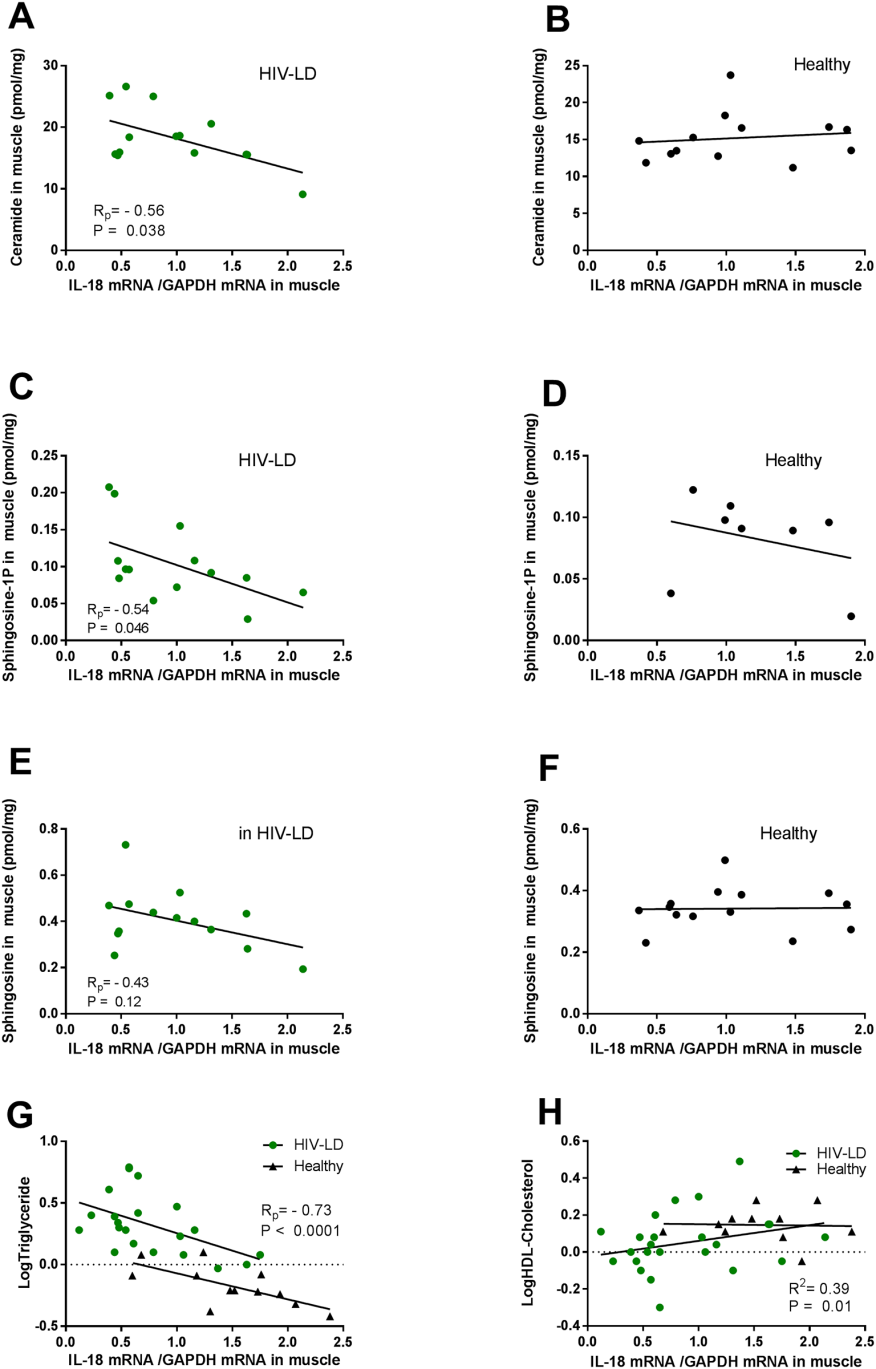
The correlation relationship between muscle IL-18 mRNA and muscle sphingolipid content, circulating triglycerides and HDL-cholesterol in patients with HIV-lipodystrophy (to the right) and in healthy controls (to the left). IL-18 mRNA in muscle is negatively correlated to ceramide (A) and sphingosine-1P (C) content in muscle in patients with HIV-Lipodystrophy, but not in healthy controls (B, D, F). Il-18 mRNA in muscle is negatively correlated to triglycerides in patients with HIV-Lipodystrophy and in healthy controls (G), and positively correlated to HDL-Cholesterol in patients with HIV-lipodystrophy. Regressions lines, correlations coefficient and significance levels are given for healthy controls and patients with HIV-Lipodystrophy separately.

In patients with HIV-lipodystrophy, low expression of IL-18 mRNA in skeletal muscle correlated to high levels of ceramides (R_p_ = -0.56; p = 0.038) (Fig 3A) and high levels of sphingosine-1P (IL-18 mRNA R_p_ = -0.54, p = 0.046) (Fig 3C). The same trend was observed for sphingosine (R_p_ = -0.43, p = 0.12) (Fig 3E). In healthy subjects, IL-18 mRNA in skeletal muscle did not correlate to muscle sphingolipids (Fig 3B, 3D and 3F), although the correlation for sphingosine-1P only included 8 samples and a possible correlation may be lost due to low n-value.

Reduced expression of IL-18 mRNA in skeletal muscle was associated with increased levels of circulating triglycerides in all subjects pooled together (R_p_ = -0.73, p<0.0001) (Fig 3G). The same observation was found for IL-18R mRNA expression in skeletal muscle (R_p_ = -0.56, p = 0.0004, data not shown). When patients with HIV-lipodystrophy and healthy subjects were analysed individually, the same correlation between IL-18 mRNA and triglycerides remained (HIV-LD R_p_ = -0.51, p = 0.02; Healthy subjects R_p_ = -0.69, p = 0.014). Reduced expression of IL-18 mRNA and IL-18R mRNA in skeletal muscle was also associated with reduced levels of HDL-cholesterol in all subjects together (for IL-18 mRNA: Rp = 0.39, p = 0.01; for IL-18R mRNA: Rp = 0.48, p = 0.002) (Fig 3H) but not when the groups were analysed separately. No correlations were found between total-cholesterol or LDL-cholesterol and IL-18 mRNA (data not shown).

As patients with HIV-lipodystrophy had a lower VO_2_ max than the healthy controls in group 2 we examined if the increased levels of sphingolipids were related to low levels of VO_2_ max in patients with HIV-lipodystrophy. There was, however, no correlation between sphingolipids and VO_2_max/kg (ceramide R_p_ = 0.23, p = 0.42; Sphingosine-1P R_p_ = 0.20, p = 0.48; sphingosine R_p_ = 0.37, p = 0.19).

In addition, no correlation was found between muscle IL-18 mRNA expression and fat distribution (BMI, fat mass, limb or trunk fat mass) in healthy controls or in patients with HIV-lipodystrophy when analysed separately (data not shown).

No correlation was found between muscle IL-18 mRNA expression and glucose metabolism (plasma insulin, HOMA, glucose and insulin response during an OGTT) in healthy controls or in patients with HIV-lipodystrophy when analysed separately (data not shown).

### Expression of mitochondrial genes and fatty acid transporters in skeletal muscles and correlation to ceramide content in skeletal muscle

**Fig 4:**
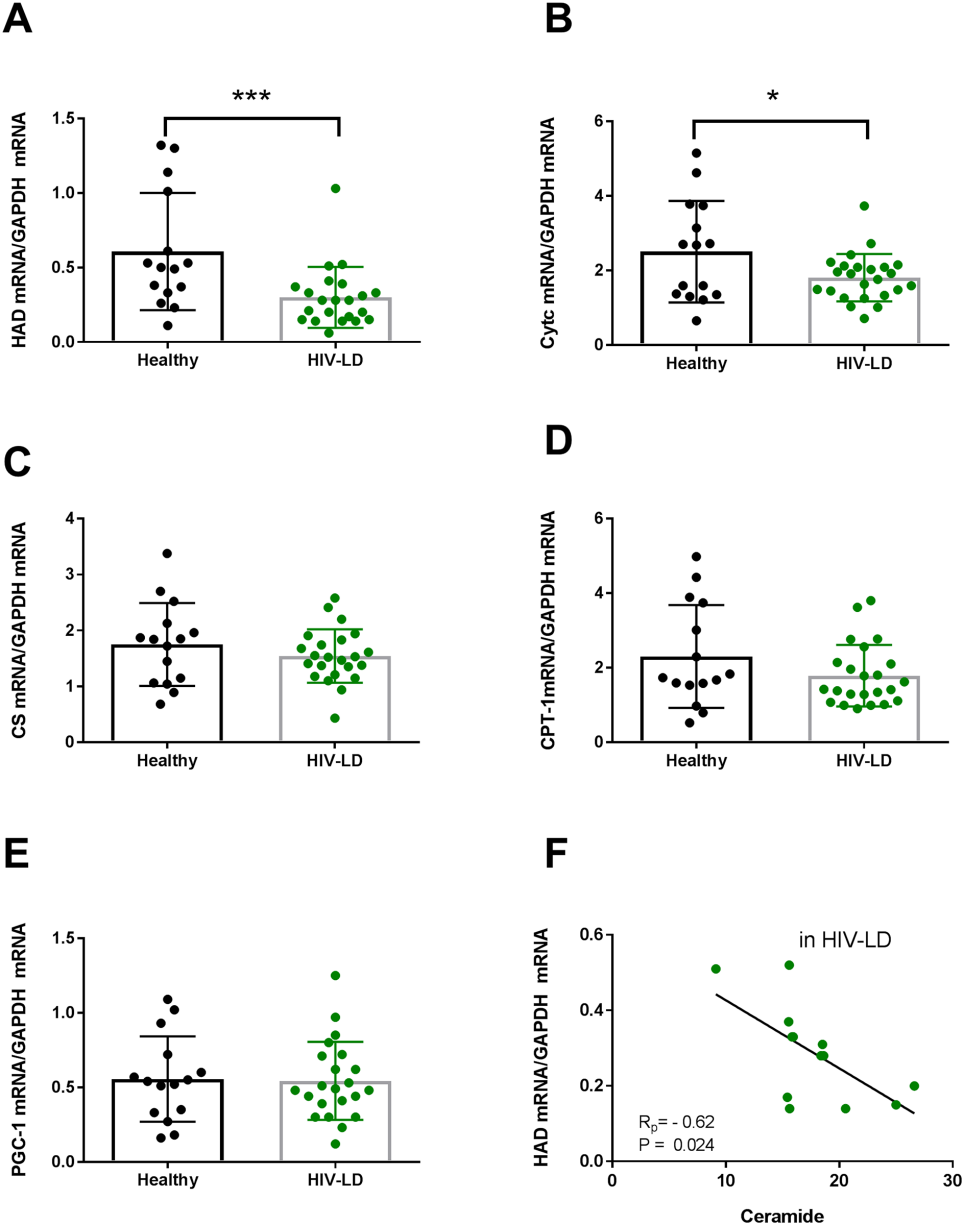
Patients with HIV-Lipodystrophy have reduced levels of HAD mRNA (A) and Cytochrome c mRNA (B) in skeletal muscle, but no difference in citrate synthase mRNA (C), CPT-1 mRNA (D) and PGC-1 mRNA (E). HAD mRNA correlated negatively to the ceramide content (F). The levels of genes were calculated with GAPDH as a housekeeping gene. In the dot plots data for each subjects are given and the line represent means and SD. * P<0.05 and ***P<0.001 for healthy vs HIV-lipodystrophy patients. Regressions lines are given HIV-patients separately.

Patients with HIV-lipodystrophy displayed lower mRNA expressions of β-hydroxy acyl-CoA dehydrogenase (β-HAD) (p = 0.004) (Fig 4A) and cytochrome c oxidase (p = 0.03) (Fig 4B) compared to controls. No differences between groups were observed for citrate synthase, CPT-1, and PGC-1 alpha (Fig 4C, 4D and 4E).

In patients with HIV-Lipodystrophy the content of ceramide in muscle correlated negative to the levels of β-HAD mRNA expressions (R = -0.61; p = 0.02) (Fig 4F), but not to cytochrome c oxidase (R = -0.10, p = 0.72) (Data not shown).

## Discussion

The major finding of this study is that the expression of IL-18 mRNA and IL-18 receptor mRNA is reduced in skeletal muscle in patients with HIV-lipodystrophy compared to healthy age-matched men. Furthermore, low expression of muscle IL-18 mRNA correlates to high levels of ceramides in skeletal muscle and to increased levels of circulating triglycerides and low levels of HDL-cholesterol in patients with HIV-lipodystrophy.

### IL-18 and IL-18 receptor in skeletal muscle

IL-18 is expressed in skeletal muscle [5] and high expression of IL-18 has been reported in skeletal muscle in inflammatory diseases such as inflammatory myopathies [48] and COPD [49]. Macrophages and dendritic cells are suggested to be the main producers of IL-18 [48]. Furthermore, it has been suggested that inflammation e.g. TNF-α trigger the IL-18 expression in skeletal muscle [50].

In contrast to those studies, we found a reduced expression of IL-18 and IL-18 Receptor mRNA in skeletal muscles in patients with HIV-lipodystrophy compared to healthy controls, although circulating IL-18 was increased in those patients. Therefore, we conclude that IL-18 signaling pathway is impaired in skeletal muscle in patients with HIV-lipodystrophy and that the muscle cell is not a source of circulating IL-18. Instead, we suggest that in healthy humans IL-18 is working in a local manner in skeletal muscle. It has previously been demonstrated that IL-18 mRNA is induced e.g. by TNF without influence circulating IL-18 [50]. Furthermore, several proteins (e.g. IL-8 and BDNF) are produced by skeletal muscle but not released into the circulation, and those proteins work via autocrine or paracrine mechanisms, exerting their effects on signalling pathways within the muscle itself [51].

The explanation for reduced IL-18 and IL-18 receptor expression in skeletal muscle is intriguing as the underlying mechanisms for the IL-18 production and IL-18 receptor regulation are still poorly understood. IL-18 production is tightly regulated, and achieved in a caspase-1 dependent or in a caspase-1-independent way. The components for cleavage of caspase-1, the inflammasome complexes, such as nucleotide-binding oligomerization domain receptors 1 (NLRP)-1, NLRP-3 and NLRC-4, is present in adipose tissue [52], and in skeletal muscle [53]. In adipose tissue, NLRP1 is an innate immune sensor that functions in the context of metabolic stress to produce IL-18, preventing obesity and diet-induced metabolic dysfunction [22,23]. NLRP1 is also highly expressed in skeletal muscle in humans [54], but it is unknown how NLRP1 is regulated in skeletal muscle and if NLRP-1 stimulates production of IL-18 in skeletal muscle, in a similar way as in adipose tissue.

The reduced IL-18 and IL-18 receptor in skeletal muscle in patients with HIV-Lipodystrophy may also be link to IL-18 resistance. Obese subjects and person with type 2 diabetes demonstrate impaired IL-18 responsiveness in leucocyte despite increased circulating levels of IL-18 [55]. The IL-18 receptor is suggested to be the responsible molecular site for the observed IL-18 resistance, as leucocyte from obese and type 2 diabetics demonstrated a reduced IL-18 receptor expression on leucocyte. However, no mechanism for reduced IL-18 receptor expression was found and the expression of IL-18 on the leucocyte was not measured. Netea and colleagues [55] explains IL-18 resistance as an immunological phenomenon; and hypothesize that a similar resistance to the metabolic effect of IL-18 is also present [55]. It is possible that the reduced IL-18 receptor expression in skeletal muscle found in the current study play a role in a resistance to the metabolic effects of IL-18. This has been shown for IL-6. where skeletal muscle in obesity-associated type 2 diabetes develops a resistance to IL-6 [56]. However, this hypothesis has to be tested an in vitro study.

### The role of IL-18 in lipid metabolism

In the last years, several animal studies have demonstrated that IL-18 play a role in metabolism, which is independent of its inflammatory role. Mice lacking IL-18 or the IL-18 receptor and therefore impaired IL-18 signalling become obese and display hyperinsulinemia, insulin resistance and dyslipidemia [19–21]. We found an inverse correlation between IL-18 expression in skeletal muscle and systemic triglycerides and HDL-cholesterol in both healthy subjects and in patients with HIV-lipodystrophy. Production of VLDL-TG is in part due to be the increased flux of FFA to the liver in combination with insulin resistance associated hyperinsulinemia [57,58] and this may be a way linking the role of IL-18 in skeletal muscle to dyslipidemia.

In addition, IL-18 and IL-18 receptor deficient mice have an excess of intramyocellular lipids (IMCL) which in part is explained by impaired beta-oxidation in muscle due to a defect in one of major pathway that regulates fatty acid oxidation AMP-activated protein kinase (AMPK) [21]. Treating myotubes or skeletal muscle strips with IL-18 activates AMPK and increases fat oxidation. Furthermore, overexpression of IL-18 in mice leads to reduced fat mass, increased activation of AMPK and increased mRNA abundance of β-hydroxyacyl-CoA-dehydrogenase (HAD), a key enzyme involved in mitochondrial function and hence increased fat oxidation [21] -, implicating IL-18 in metabolic homeostasis.

Having found that IL-18 mRNA expression was lower in skeletal muscle in patients with HIV-lipodystrophy, we investigated whether this was associated to an increased content of IMCL, as observed in mice lacking the IL-18 signalling pathway [21]. It is known that patients with HIV-lipodystrophy have increased level of IMCLs [24,30–32]. IMCLs are mainly composed by triacylglycerol but also include lipid intermediates such as diacylglycerol, sphingolipid, and phospholipid. Sphingolipids, including ceramide, sphingosine, sphingosine-1P and sphinganine have never been measured in skeletal muscle from patients with HIV-lipodystrophy, but are altered in other metabolic state such as obesity and type 2 diabetes. [59–61]. We found a trend towards increased contents of the sphingolipids ceramide and sphingosine, and that the reduced levels of IL-18 mRNA in muscle correlated with increased contents of ceramides and sphingosine-1P in patients with HIV-lipodystrophy, but not in healthy controls, demonstrating that skeletal muscle IL-18 and sphingolipids are linked. As this study is a cross-sectional study it is not possible to describe the causal relationship. Ceramide has been demonstrated to be involved in IL-18 production in macrophages and adipose tissue via increased production of reactive oxygen species, which acts as a secondary signal for NLRP3 activation leading to induction of caspase-1 cleavage and thereby increased IL-18 [62]. However, in the current study IL-18 expression was decreased and not increased in skeletal muscle in HIV-lipodystrophy, and ceramide can therefore not explain reduced expression of IL-18. In contrast, it is possible that low expression of IL-18 is causal involved in elevated content of ceramide, just like in the IL-18 deficient and IL-18 receptor deficient mice.

It is unknown whether increased level of ceramide is directly a cause of HAART, as no studies have demonstrated an effect of HAART *per se* on ceramide. Instead, hiv proteins (gp120 og TAT) can induces sphingolipids [63]. In our patients viral load was below the detection limits for all patients therefore it is unlikely that the increased levels of ceramide in muscle may be induced by hiv *per se*. Instead, ectopic lipid deposition is a part of the lipodystrophy syndrome seen in HAART treated HIV patients, and therefore the increased ceramide levels may be indirectly results of the antiretroviral therapy.

### IL-18 and mitochondrial activity

As described in rodents, IL-18 increases fatty acid oxidation through activation of AMPK and mitochondrial oxidation [21]. Patients with HIV-lipodystrophy display impaired fat oxidation [30] [29] and two recent studies show impaired mitochondrial oxidative phosphorylation [64,65] and activity of enzymes involved in fat oxidation; e.g. β-HAD and citrate synthase [33] in skeletal muscle in HIV patients. Our data is in accordance with those findings as we found reduced expression of β-HAD and cytochrome c oxidase mRNA in skeletal muscle in HIV patients with lipodystrophy. Furthermore, we showed that the ceramide content is negatively correlated with the expression of β-HAD. It has previously been demonstrated that ceramides alter mitochondrial dynamics in skeletal muscle [41] and therefore, impaired mitochondrial function may be secondary to increased ceramide observed in skeletal muscle in patients with HIV-LD. However, impaired mitochondrial fatty acid oxidation is also known to increase IMCLs, diacylglycerols, and ceramides [66].

As specific mRNA measurements correlated to each due to the use of the same house keeping gene (GAPDH) it was not possible to make a correlational relationship analysis between IL-18 mRNA expression and mRNA expression of mitochondrial gene in our study. As a negative correlation between ceramide content and the expression of β-HAD mRNA as well as a negative correlation between ceramide content and the expression of IL-18 mRNA were present, we can only speculate that IL-18 mRNA may correlate to expression of mitochondrial genes as well.

In conclusion, our findings suggest that muscular IL-18 may be involved in the regulation of intramuscular lipid metabolism and hypertriglyceridemia.
