# Correction: Low expression of IL-18 and IL-18 receptor in human skeletal muscle is associated with systemic and intramuscular lipid metabolism—Role of HIV lipodystrophy

**DOI:** 10.1371/journal.pone.0196241

**Published:** 2018-04-17

**Authors:** Birgitte Lindegaard, Thine Hvid, Helene Wolsk Mygind, Ole Hartvig Mortensen, Thomas Grøndal, Julie Abildgaard, Jan Gerstoft, Bente Klarlund Pedersen, Marcin Baranowski

The fourth author’s name is incorrect. The correct name is: Ole Hartvig Mortensen. The correct citation is: Lindegaard B, Hvid T, Wolsk Mygind H, Mortensen OH, Grøndal T, Abildgaard J, et al. (2018) Low expression of IL-18 and IL-18 receptor in human skeletal muscle is associated with systemic and intramuscular lipid metabolism—Role of HIV lipodystrophy. PLoS ONE 13(1): e0186755. https://doi.org/10.1371/journal.pone.0186755
